# Enhanced MYC association with the NuA4 histone acetyltransferase complex mediated by the adenovirus E1A N-terminal domain activates a subset of MYC target genes highly expressed in cancer cells

**DOI:** 10.18632/genesandcancer.160

**Published:** 2017-11

**Authors:** Ling-Jun Zhao, Paul M. Loewenstein, Maurice Green

**Affiliations:** ^1^ Department of Microbiology and Molecular Immunology/Institute for Molecular Virology, Saint Louis University School of Medicine, Doisy Research Center, St. Louis, Missouri, USA

**Keywords:** RNA-seq, MYC (c-Myc), histone acetyltransferase complex, E1A binding protein p300 (P300), cancer, E1A 1-80, TRRAP, NuA4 complex, ribosome biogenesis

## Abstract

The proto-oncogene MYC is a transcription factor over-expressed in many cancers and required for cell survival. Its function is regulated by histone acetyltransferase (HAT) complexes, such as the GCN5 complex and the NuA4/Tip60 complex. However, the roles of the HAT complexes during MYC function in cancer have not been well characterized. We recently showed that adenovirus E1A and its N-terminal 80 aa region, E1A 1-80, interact with the NuA4 complex, through the E1A TRRAP-targeting (ET) domain, and enhance MYC association with the NuA4 complex. We show here that the ET domain mainly targets the MYC-NuA4 complex. By global gene expression analysis using E1A 1-80 and deletion mutants, we have identified a panel of genes activated by targeting the MYC-NuA4 complex and notably enriched for genes involved in ribosome biogenesis and gene expression. A second panel of genes is activated by E1A 1-80 targeting of both the MYC-NuA4 complex and p300, and is enriched for genes involved in DNA replication and cell cycle processes. Both panels of genes are highly expressed in cancer cells. Since the ET domain is essential for E1A-mediated cellular transformation, our results suggest that MYC and the NuA4 complex function cooperatively in cell transformation and cancer.

## INTRODUCTION

Adenovirus E1A 243R (E1A) is a viral oncoprotein that transforms cells by targeting several key cellular factors including p300, TRRAP, and Rb [1, 2]. E1A targets TRRAP via an N-terminal domain; however, the functional consequence of E1A-TRRAP association remains unclear. E1A 1-80, the E1A N-terminal 80 aa region, represses transcription of some viral and cellular promoters *in vivo* and *in vitro* [3–6]. E1A 1-80 targets p300 and TRRAP [7]. By proteomic and co- immunoprecipitation analyses, we recently showed that E1A 1-80 and the full-length E1A interact with the NuA4 complex and enhance its association with MYC [7]. This activity requires the E1A N-terminal TRRAP-targeting (ET) domain [7]. The ET domain is capable of enhancing MYC association with the NuA4 complex when fused to the N-terminus of MYC [8]. Since association with TRRAP is essential for transformation by E1A and by MYC [9–11], we hypothesize that MYC association with the NuA4 complex may be increased during cellular proliferation and transformation induced by MYC and by E1A.

The proto-oncogene MYC is over-expressed in many cancers and required by essentially all cancers for survival [12–14]. MYC over-expression promotes tumorigenesis [14–16], and transient down-regulation of MYC below a certain level causes tumor regression in animal models [17]. Histone acetyltransferase (HAT) complexes, such as the NuA4 complex and the GCN5 complex, participate in transcriptional activation by MYC [18–21]. However, how the HAT complexes participate in MYC function during cancer development remains uncharacterized. The human NuA4 complex, the largest HAT complex containing up to 20 subunits [22], has a core HAT enzyme Tip60, and is involved in chromatin remodeling, gene activation, and DNA damage repair [22–24]. Proteomic analysis of MYC suggests that MYC associates with the NuA4 complex in mouse embryonic stem cells [21]. MYC and some components of the NuA4 complex are found to co-occupy a set of promoters and co-activate those genes (“MYC-module” genes) in mouse embryonic stem cells to help define stem cell characteristics [21]. Significant over-expression of a similar set of genes in human cancers seems to be correlated with tumor invasiveness, suggesting the importance of MYC association with the NuA4 complex in cancer [21].

In this report, we performed RNA-seq analysis with human foreskin fibroblast cells (HS68) expressing E1A 1-80 and mutants lacking a functional ET domain or p300- targeting domain, and identified two panels of genes that are activated by E1A 1-80 targeting of the MYC-NuA4 complex or by targeting of both the MYC-NuA4 complex and p300. Expression of both panels of genes is increased in three cancer cell lines examined compared to HS68 cells, suggesting that expression of both panels of genes in cancer may involve MYC association with the NuA4 complex.

## RESULTS AND DISCUSSION

### Activation of MYC target genes by E1A 1-80 correlates with enhanced MYC association with the NuA4 complex

**Figure 1:**
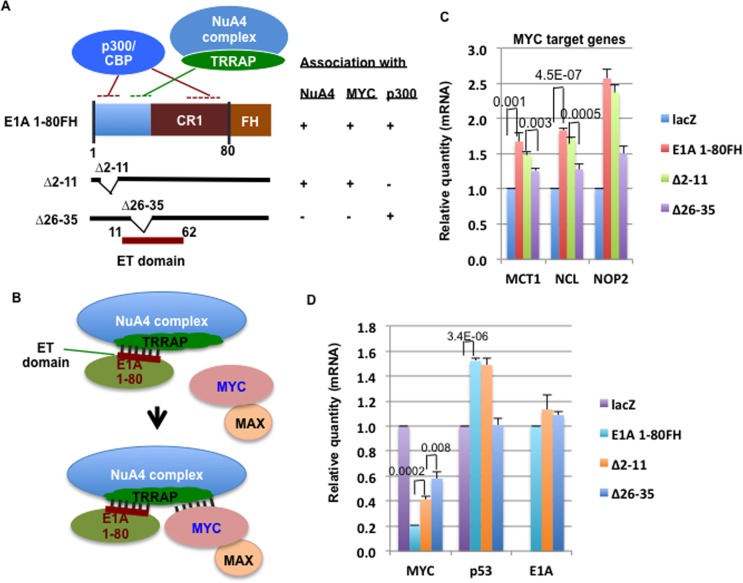
E1A TRRAP-targeting (ET) domain is essential for enhancement of MYC association with the NuA4 complex and for E1A 1-80 activation of selected MYC target genes A. Illustration of E1A 1-80FH expression construct and E1A 1-80 functional domains. The ET domain is essential for E1A 1-80 interaction with the NuA4 complex and enhancement of MYC association with this complex [7]. B. Model for E1A 1-80 enhanced MYC association with the NuA4 complex. The ET domain interacts with TRRAP within the NuA4 complex, and facilitates association of MYC/MAX with this complex. C. E1A 1-80 activates MYC target genes MCT, NCL, and NOP2. RT-qPCR analysis was performed with gene specific primers (Table S1), using GAPDH as internal control. Graph represents average RT-qPCR results of the three sets of experiments with standard deviation shown. Both E1A 1-80FH and ∆2-11 activated these genes, whereas ∆26-35 had significantly lower effects. Numbers on top of bars are P values from two-tailed student *t* test. D. E1A 1-80 represses MYC and activates TP53. RT-qPCR analysis was under the same conditions of C. Both E1A 1-80FH and ∆2-11 activated TP53, while all three E1A 1-80 constructs strongly repressed MYC. Expression of the three E1A 1-80 constructs was at similar levels. Numbers on top of bars are P values from two-tailed student *t* test. Only pairs with a fold change of less than 2 were analyzed.

We have previously shown that both the full length E1A and its N-terminal 80 aa (E1A 1-80) enhance MYC association with the NuA4 complex [7]. Further, we have shown that E1A 1-80 with deletion of aa 2-11, which inactivates p300 targeting remains capable of interaction with the NuA4 complex, whereas deletion of aa 26-35, which inactivates the E1A TRRAP-targeting (ET) domain abolishes E1A 1-80 interaction with the NuA4 complex (for a summary, see Figure 1A). These data suggest that the ET domain interacts with TRRAP in the NuA4 complex to promote its association with MYC (Figure 1B).

Because E1A 1-80 enhances MYC association with the NuA4 complex through the ET domain, it is reasonable to expect that TRRAP-targeting helps assemble a MYC-NuA4 complex that activates a subset of MYC target genes. To examine this possibility, we designed a strategy for examination of MYC target gene activation using contact-inhibited human fibroblast cells (HS68). In culture, HS68 cells become contact-inhibited after forming a confluent monolayer, possibly resulting in down-regulation of genes important for cell proliferation. However, they resume growth and proliferation after re- plating and have been used to examine MYC regulation of gene expression [8].

HS68 cells were infected with Ad viral expression vectors for E1A 1-80FH (Flag-HA tag on the C-terminus of E1A 1-80), and two deletion mutants, ∆2-11 and ∆26-35 which do not interact with p300 or the MYC-NuA4 complex, respectively (Figure 1A) [7]. To examine potential function of E1A 1-80 through MYC, RT-qPCR analysis was performed with gene-specific primers for three selected genes that are known to be activated by MYC: MCT1 (monocarboxylate transporter 1), NCL (nucleolin), and NOP2 (nucleolar protein 2 homologue) [8]. As shown in Figure 1C, both E1A 1-80FH and ∆2-11 activated all three genes, while ∆26-35 was defective. Thus, activation of these MYC target genes is correlated with a functional ET domain which promotes MYC association with the NuA4 complex (Figure 1A). Consistent with previous results [25], expression of E1A 1-80FH or the two deletion mutants repressed cellular MYC mRNA (Figure 1D - MYC). Since E1A 1-80FH repressed MYC mRNA more strongly than either ∆2-11 or ∆26-35, it is possible that targeting of the MYC- NuA4 complex and targeting of p300 are both involved in this repression. However, statistical analysis suggested that ∆2-11 was significantly more effective than ∆26-35 in repression of MYC. Both E1A 1-80FH and ∆2-11 significantly activated TP53 (Figure 1D - TP53), consistent with the observation that over-activation of MYC usually leads to TP53 activation [26, 27]. In contrast, ∆26-35 did not activate TP53, possibly because it lacks a functional ET domain to enhance MYC association with the NuA4 complex. Analysis of the mRNA for E1A 1-80FH and its two deletion mutants showed that all forms of E1A 1-80FH are expressed to comparable levels (Figure 1D - E1A).

### The ET domain targets mainly the NuA4 complex

Since the ET domain interacts with TRRAP that is present in at least three HAT complexes [22], it remains unclear as to which HAT complex the ET domain interacts with. Therefore, we constructed an ET-MYC fusion protein that efficiently associates with components of the NuA4 complex by co-IP and Western blot analysis [8]. Since the NuA4 complex contains up to 20 subunits, we examined ET-MYC interaction with the NuA4 complex more thoroughly by a proteomic approach. Lentivirus vectors were used to express i) FH-MYC to detect interaction with the NuA4 complex, ii) ET-MYC to detect enhanced association with the NuA4 complex, and iii) Flag-Tip60 to detect components of the NuA4 complex as control. Cell lysates were immunoprecipitated with Flag antibody beads, and bound protein complexes eluted with Flag peptide and subjected to liquid chromatography- tandem MS analysis and associated proteins identified. As shown in Table 1, this analysis shows that ET-MYC associates with 14 known components of the NuA4 complex, while FH-MYC associates with only three. ET- MYC associated proteins overlap with those associated with Tip60 (control for the NuA4 complex). Thus, the ET- MYC complex closely resembles a MYC-NuA4 complex. Importantly, from this analysis and our previous proteomic analysis [7], the ET domain appears to associate only with the NuA4 complex, and not the GCN5 or PCAF complex that also contains TRRAP [22], suggesting a key role for the NuA4 complex in the function of the ET domain.

**Table 1:** proteins interacting with ET-MYC (identified by proteomic analysis)

			# of identified peptides			
Identified Proteins	Protein Name	Mol Wt	GFP	MYC	ET-MYC*	Tip60
Transformation/transcription domain-associated protein	TRRAP	436 kDa	0	0	52	17
Histone acetyltransferase KAT5	KAT5 (Tip60)	59 kDa	0	0	3	20
E1A-binding protein p400	EP400	343 kDa	0	0	26	12
RuvB-like 1	RUVB1	50 kDa	1	5	14	9
RuvB-like 2	RUVB2	51 kDa	0	2	18	5
Actin-like protein 6A	ACL6A	47 kDa	0	1	8	3
DNA methyltransferase 1-associated protein 1	DMAP1	53 kDa	0	0	8	4
Bromodomain-containing protein 8	BRD8	95 kDa	0	0	4	4
Inhibitor of growth protein 3	ING3	45 kDa	0	0	1	3
Chromatin modification-related protein MEAF6	MEAF6	22 kDa	0	0	2	2
MRG/MORF4L-binding protein	MRGBP	22 kDa	0	0	1	1
YEATS domain-containing protein 4	YEATS4	27 kDa	0	0	2	1
Enhancer of polycomb homolog 1	EPC1	93 kDa	0	0	2	0
Enhancer of polycomb homolog 2	EPC2	91 kDa	0	0	2	0
Myc proto-oncogene protein	MYC	50 kDa	0	5	7	0
Protein MAX	MAX	11 kDa	0	1	1	0
MAX gene-associated protein	MGA	332 kDa	0	1	1	0
Ribonucleoside-diphosphate reductase large subunit	RIR1	90 kDa	0	0	0	21
Unconventional myosin-Ic	F5H6E2	119 kDa	1	0	0	9
cAMP-dependent protein kinase type I-alpha regulatory	K7EM13	17 kDa	0	3	3	0
Keratin, type II cytoskeletal 1	K2C1	66 kDa	0	0	3	2
Vacuolar protein sorting-associated protein 72 homolog	VPS72	41 kDa	0	0	3	1
E3 ubiquitin-protein ligase HUWE1	HUWE1	482 kDa	0	0	1	2
Protein PRRC2C	E7EPN9	309 kDa	0	0	2	0
Protein phosphatase 1 regulatory subunit 12A	F8VZN8	77 kDa	0	0	0	3
Merlin	MERL	70 kDa	0	0	0	2
Septin 10, isoform CRA_c	B5ME97	63 kDa	0	0	0	2
26S protease regulatory subunit 8 (Fragment)	J3QSA9	29 kDa	0	0	2	0
Heat shock protein HSP 90-alpha	HS90A	85 kDa	0	0	0	3
Juxtaposed with another zinc finger protein 1	JAZF1	27 kDa	0	0	2	0
26S proteasome non-ATPase regulatory subunit 11	PSD11	47 kDa	0	0	0	2
PRKC apoptosis WT1 regulator protein	PAWR	37 kDa	0	0	0	2

* Orange color indicates components of the NuA4 complex; blue color MYC-related proteins.

### E1A 1-80 modulates global gene expression by targeting the MYC-NuA4 complex and p300 separately and cooperatively

E1A 1-80 contains the domains of the full-length E1A that target the HAT enzyme p300 and the MYC-NuA4 HAT complex (Figure 1A). To identify genes activated by E1A 1-80 targeting of the these two HAT activities, RNA-seq analysis was performed with mRNAs from HS68 cells infected with adenovirus vectors expressing control lacZ, E1A 1-80FH, and the two deletion mutants, ∆2-11 and ∆26-35. Importantly, the ∆2-11 mutant remains capable of interaction with TRRAP and therefore the NuA4 complex (see Figure 1A) in the absence of potential interference from p300-targeting, and thus may help reveal genes activated by the enhanced MYC association with the NuA4 complex. The ∆26-35, on the other hand, may regulate gene expression by interaction with p300 as one of the major cellular targets for E1A.

**Figure 2:**
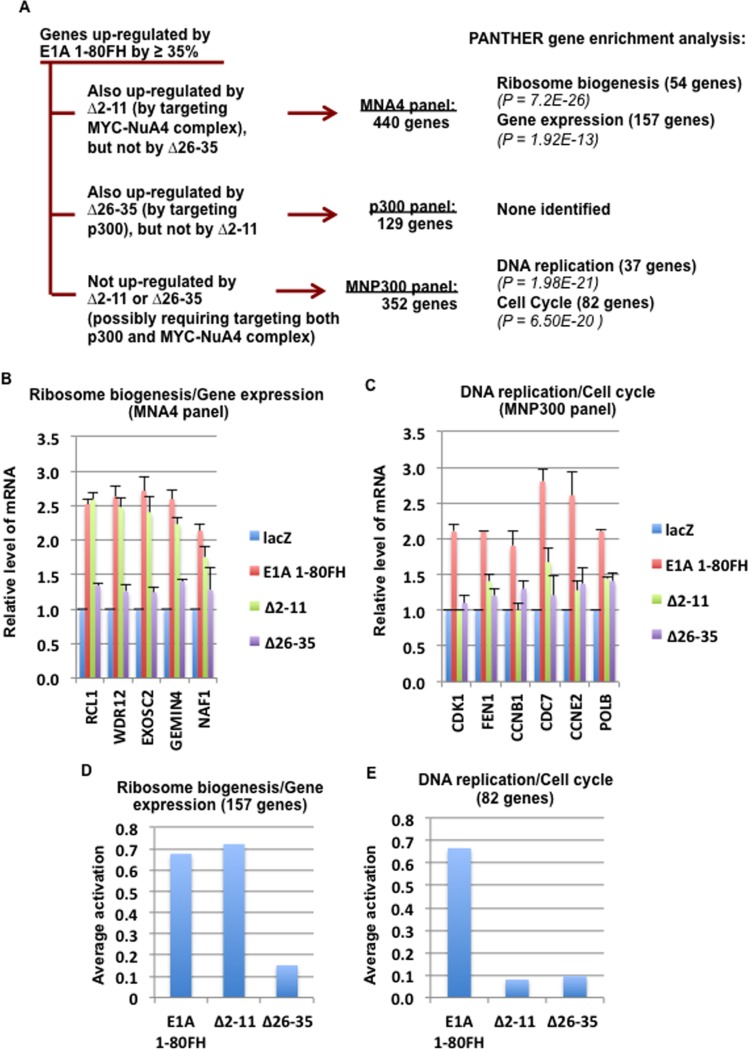
E1A 1-80 activates three panels of genes by targeting the MYC-NuA4 complex and p300 separately and cooperatively A. Activation of three panels of genes within those activated by E1A 1-80FH identified by RNA-seq analysis. The MNA4 panel genes are defined by three criteria: i) activation by E1A 1-80FH of ≥ 35%, and ii) a ratio of activation by ∆2-11 /activation by E1A 1-80FH of ≥ 65%, and iii) a ratio of activation by ∆26-35/activation by ∆2-11 of ≤ 40%. The p300 panel genes are defined similarly and overlap between activation by ∆26-35 and activation by E1A 1-80FH. The MNP300 panel genes are defined by activation of ≥ 35% by E1A 1-80FH, and an activation ratio of ≤ 40% for both ∆2-11 (∆2-11 activation/E1A 1-80FH activation) and for ∆26-35 (∆26-35 activation/ E1A 1-80FH activation). PANTHER (version 12) gene enrichment analysis [32–34] was performed online (http://www.geneontology.org). B. Activation of selected MNA4 panel genes involved in ribosome biogenesis/gene expression by RT-qPCR analysis. The three sets of RNA used for RNA-seq analysis were used for RT-qPCR analysis with gene specific primers. Graph represents average RT-qPCR results from the three sets of RNA samples with standard deviations shown. C. Activation of selected MNP300 panel genes involved in DNA replication/cell cycle by E1A 1-80FH. These genes were activated by ∆2-11 and ∆26-35 only poorly. Conditions were the same as in B. D. Average activation of genes involved in ribosome biogenesis/gene expression from the MNA4 panel (157 genes total). Data were derived from RNA-seq results. E. Average activation of genes involved in DNA replication/cell cycle from the MNP300 panel (82 genes total).

RNA-seq analysis revealed more than 2000 genes activated over 35% by E1A 1-80FH (with a P value ≤ 0.05). Three panels of genes are derived from these E1A 1-80-activated genes (Figure 2A): i) those that overlap with genes activated by ∆2-11 and require targeting of the MYC-NuA4 complex (MYC-NuA4 panel, or MNA4 panel); ii) those that overlap with genes activated by ∆26-35 and require targeting of p300 (p300 panel), and iii) genes that are only weakly activated by both ∆2-11 and ∆26-35 and therefore require targeting of both the MYC- NuA4 complex and p300 for activation (MYC-NuA4 and p300 panel, or MNP300 panel). Since E1A 1-80 region has been shown to interact with factors in addition to p300 and the NuA4 complex (which contains TRRAP, p400 and other subunits) [2], it remains to be determined if any of these additional factors contributes to the activation of these >2000 identified genes.

**Figure 3:**
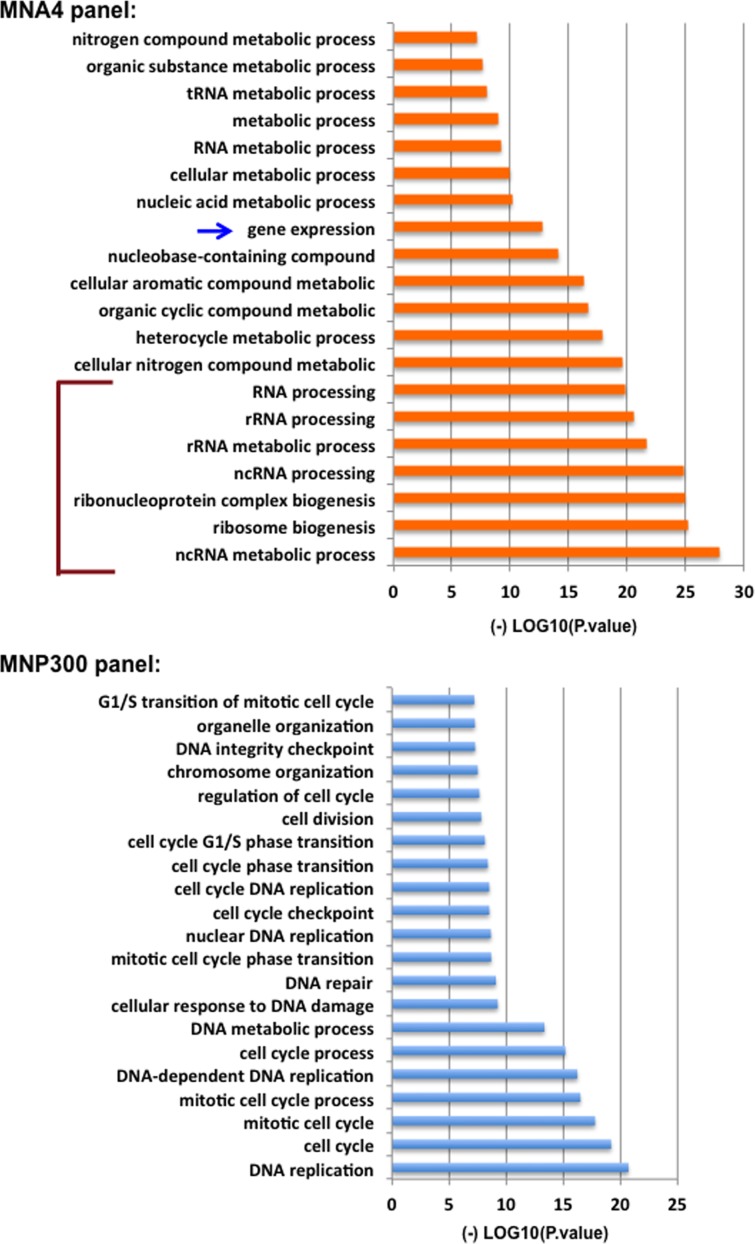
PANTHER gene enrichment analysis of the MNA4 panel and MNP300 panel genes Analysis was performed online (http://www.geneontology.org). Biological processes with a P value of < 1×10-7 are shown. Biological processes indicated by the bracket and red arrows are closely related to ribosome biogenesis. “Gene expression” is marked with a blue arrow.

To understand the function of these different panels of genes, PANTHER analysis for gene enrichment in biological processes was performed (see Figure 2A legend). This analysis revealed that the MNA4 panel genes (440 genes total) are strongly enriched for genes involved in gene expression and ribosome biogenesis (Figure 2A and Figure 3), while the p300 panel genes (129 genes total) do not enrich for any identifiable biological processes. The MNP300 panel genes (352 genes total) are most significantly enriched in genes involved in DNA replication and cell cycle (Figure 2A and Figure 3).

To confirm activation of the identified genes by E1A 1-80, selected MNA4 panel genes involved in ribosome biogenesis/gene expression and MNP300 panel genes involved in DNA replication/cell cycle were examined by RT-qPCR. As shown (Figure 2B), the selected MNA4 panel genes involved in ribosome biogenesis/gene expression are activated efficiently by both E1A 1-80FH and ∆2-11 but not by ∆26-35. These results confirm that E1A 1-80 activates the MNA4 panel genes through the ET domain by targeting the NuA4 complex and enhancing its association with MYC (Figure 1B). The selected MNP300 panel genes involved in DNA replication/cell cycle are activated by E1A 1-80FH much more efficiently than by ∆2-11 or by ∆26-35 (Figure 2C), confirming that activation of the MNP300 panel genes involves targeting of both the MYC-NuA4 complex and p300.

We next analyzed the average activation of all the MNA4 panel genes involved in ribosome biogenesis/gene expression (157 total out of the MNA4 panel), utilizing the data from the RNA-seq analysis. This analysis showed that both E1A 1-80FH and ∆2-11 activate this group of genes on average by > 60%, whereas ∆26-35 is defective in activation (Figure 2D). In contrast, the MNP300 panel genes involved in DNA replication/cell cycle (82 total out of the MNP300 panel) were activated by E1A 1-80FH (by ~ 65%), but poorly by both ∆2-11 and ∆26-35 (by < 10%, Figure 2E), confirming that both the MYC-NuA4 complex and p300 are involved in activation of the MNP300 panel genes.

### The MNA4 panel genes are authentic MYC target genes in HS68 cells

The 440 MNA4 panel genes (Figure 2A) were compared with the 1037 genes activated by MYC by > 20% under similar cell culture conditions [8] and 276 (63%) are found to overlap, suggesting that enhanced MYC association with the NuA4 complex promoted by E1A 1-80FH (and ∆2-11) activates a large portion of authentic MYC target genes. Other genes of the MNA4 panel may be authentic MYC target genes with lower levels of activation by MYC. In contrast, the 352 MNP300 panel genes (Figure 2A) have 21 (6%) overlapping with the same 1037 MYC activated genes, consistent with the possibility that activation of the MNP300 panel genes requires targeting of both the MYC-NuA4 complex and p300, i.e., targeting of either the MYC-NuA4 complex or p300 is insufficient for activation. Importantly, out of the 54 ribosome biogenesis genes from the MNA4 panel, 50 (93%) are activated by MYC in HS68 cells [8], consistent with their activation by MYC association with the NuA4 complex.

### The MNA4 panel and MNP300 panel genes are highly expressed in cancer cells and candidates for important function in cancer

**Figure 4:**
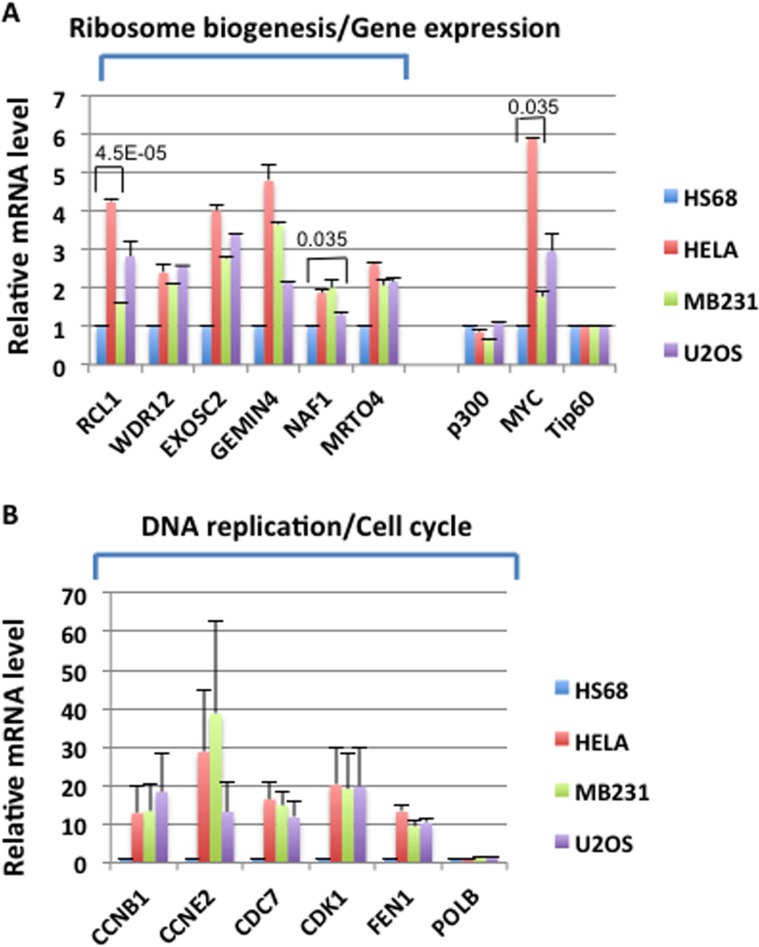
High level expression of selected MNA4 panel (A) and MNP300 panel (B) genes in cancer cell lines RNA was prepared from the indicated cell lines (all from ATCC) from sub-confluent cells, and RT-qPCR performed for the selected genes. Tip60, which had the least variation among the cell lines, was used for internal control for the qPCR reactions. Results are the average of two experiments. For comparison, expression of p300 and Tip60 is not increased in the cancer cell lines (panel A). Numbers on top of bars are P values from two-tailed student *t* test. Only pairs with a fold change of less than 2 were analyzed.

To examine the potential roles of the MYC-NuA4 complex in cancer, we first compared the expression of the MNA4 panel and MNP300 panel genes in three cancer cell lines, HeLa (cervical cancer), MB231 (breast cancer), and U2OS (osteosarcoma), relative to HS68 cells. RNA was isolated and RT-qPCR performed for i) selected MNA4 panel genes involved in ribosome biogenesis/gene expression and ii) selected MNP300 panel genes involved in DNA replication/cell cycle. As shown in Figure 4, most of these selected genes are highly expressed in the three cancer cell lines to significant degrees relative to HS68 cells, with the MNP300 panel genes (Figure 4B) over- expressed at higher levels than the MNA4 panel genes (Figure 4A). Importantly, MYC is also highly expressed in the three cancer cell lines (Figure 4A). In contrast, Tip60 and p300, both with important HAT activities, are not over-expressed in the cancer cell lines (Figure 4A). PolB from the MNP300 panel was also not over-expressed in the cancer cell lines (Figure 4B). The high expression of the genes from the MNA4 panel and MNP300 panel in cancer cell lines examined are suggestive of important roles in cancer.

Since activation of the MNP300 panel genes in HS68 cells by E1A 1-80FH requires both the MYC- NuA4 complex and p300 (Figure 2), it is likely that the MYC-NuA4 complex normally cooperates with other transcription factors for efficient activation of these genes. The lack of a strong activation of these genes by MYC in HS68 cells [8] suggests that such cooperation may not exist in normal cells. However, the high expression of the MNP300 panel genes in three cancer cell lines examined (Figure 4A) suggest that the MYC-NuA4 complex and other transcription factors may cooperate in cancer cells.

The MNA4 panel of genes is most significantly enriched for genes involved in ribosome biogenesis/ gene expression. The same conclusion was drawn from global gene expression analysis with the ET-MYC fusion protein [8], which identified an E1A-activated MYC- NuA4 panel (EMNA panel) of genes (262 genes total). Comparing the MNA4 panel from this study with the EMNA panel reveals 78 genes in common which are also significantly enriched for genes involved in ribosome biogenesis (unpublished results). Genes involved in ribosome biogenesis from the MNA4 panel may increase a basic cellular function to accommodate the need of cancer cells for increased protein synthesis. Bioinformatic search for consensus MYC binding motif CACGTG has revealed predominantly ribosome biogenesis genes in human and fly genomes [28]. Importantly, increased ribosome biogenesis function has been suggested to directly contribute to cancer development [29, 30]. Further, mice with haploinsufficient MYC have reduced ribosome biogenesis/protein translation, and a longer lifespan [31], suggesting the essential role of MYC for ribosome biogenesis. Thus, it would appear that MYC association with the NuA4 complex and its activation of genes involved in ribosome biogenesis may contribute to cancer development.

In summary, our results show that genes up- regulated by the MYC-NuA4 complex alone and together with p300 are highly expressed in cancer cells and are potential candidates for important functions in cancer.

## MATERIALS AND METHODS

### Expression constructs and proteomic analysis

Adenoviral expression constructs for E1A 1-80FH, ∆2-11 and ∆26-35, and purification of adenoviruses are described earlier [7]. Lentiviral expression constructs for FH-MYC, ET-MYC, and Flag-Tip60 are described earlier [7, 8]. Generation of lentiviruses and proteomic analyses are under conditions described [7].

### Cell culture and viral infection

Human HS68, HeLa, MB231, and U2OS are from ATCC, and cultured in DMEM (Life Technologies) supplemented with 10% fetal bovine serum and 50 U/ ml of Pen/Strep. For RNA-seq analysis, HS68 cells were cultured to confluence and maintained for three days. Cells were re-plated at 1.2 × 10^6^ cells/T75 flask, and infected with 40 PFU/cell of the adenoviral expression vector in triplicates for 18 h. RNA was prepared as described previously [25].

### RNA-seq analysis

RNA samples in triplicates were subjected to PolyA selection and RNA-seq analysis at the Washington University Genome Technology Access Center as described earlier [8].

### PANTHER gene enrichment analysis

PANTHER (version 12) gene enrichment analysis [32–34] was performed online (http://www.geneontology.org). Biological processes with Bonferroni corrected (http://www.geneontology.org) P values of smaller than a set value were shown in Figure 3.

## SUPPLEMENTARY TABLES


